# Supplementary material for: GAGE is a method for identification of plant species based on whole genome analysis and genome editing
Source: Commun Biol. 2022 Sep 10;5:947. doi: 10.1038/s42003-022-03894-9 (PMC9464240; doi:10.1038/s42003-022-03894-9)
Supplement: Supplementary file 2 — Supplementary information [file 42003_2022_3894_MOESM2_ESM.pdf]

Supplementary Information

Table S1. Off targets prediction of Cs_target1 in the genomes of *Crocus sativus* and its three adulterants

Species	Number of mismatches					
	0	1	2	3	4	5
Crocus sativus	201	13	8	0	4	61
Carthamus tinctorius	0	0	0	0	0	17
Nelumbo nucifera	0	0	0	0	14	63
Zea mays	0	0	0	0	5	26

Table S2. Mismatch sites of Cs_target1 in the genome of *Crocus sativus*

Chromosome	Position	Sequence (5'→3')	Direction of strand	Number of mismatches
chr1	365527282	TTTGTGCCTACTCTtCGTGTCCTTG	-	1
chr1	562319670	TTTGTGCCTACTCTCCGTGTCCTgG	-	1
chr1	945245496	TTTGTGCCTACTCTtCGTGTCCTTG	-	1
chr1	945453723	TTTGTGCCTACTCTCtGTGTCCTTG	-	1
chr2	16933964	TTTGTGCCTACTCTCCGTGTCCTTc	+	1
chr2	9025836	TTTGTGCCTACTCTCCGTGTCCcTG	-	1
chr3	729719	TTTGTGCCTACTCTCCGcGTCCTTG	-	1
chr3	746190	TTTGTGCCTACTCTCCGcGTCCTTG	+	1
chr6	208473188	TTTGTGCCTACgCTCCGTGTCCTTG	+	1
chr6	209771094	TTTGTGCCTACgCTCCGTGTCCTTG	+	1
chr8	127085844	TTTGTGCCTACTCTCCGTGTCaTTG	-	1
chr8	65165634	TTTGTGCCTACTCTtCGTGTCCTTG	+	1
chr8	65184893	TTTGTGCCTACTCTCtGTGTCCTTG	+	1
chr1	560187580	TTTGTGCCTACTCTtCGTGTCCTgG	-	2
chr2	50064681	TTTGTGCCcACTCTCCGTGTCCTTt	+	2
chr2	79230269	TTTGTGCCcACTCTCCGTGTCCTTt	-	2
chr3	518276986	TTTGTGCCTACTCTCCGTGTCCTgc	-	2
chr5	448736026	TTTGTGCCTACTCTagGTGTCCTTG	+	2
chr5	449535986	TTTGTGCCTACTCTggGTGTCCTTG	+	2
chr5	449948285	TTTGTGCCTACTCTagGTGTCCTTG	-	2
tig00095233_pilon	187	TTTGTGCCTACTCTCCGTGTCCTgc	-	2

The red and lowercase bases are the mismatch sites; tig00095233_pilon is a scaffold that doesn't

assemble into chromosomes.

Table S3. Targets located in the ITS2 region of five plants from different classes

Species	Target name	Target sequence	Copy number
Ricinuc communis	Rc_target1	TTTCGATACATCGAGAGGGGGGCGG	222
	Rc_target2	GCCTCGTGCATGCGGTTGGCCTAAA	224
	Rc_target3	TCGGTGGTTGTAAGACTCTCTGAAA	241
Setaria italica	Si_target1	CGCCAAAAGACACTCCCAACCCAAA	19
	Si_target2	TTTGGCTCCCCGTGCTGCAAGGCGC	20
	Si_target3	TTTGGGCTGCCGGCATAACTTGTCG	20
Ginkgo biloba	Gb_target1	TGTCCCCGTGGCGCGGTTGACTAAA	1
	Gb_target2	GAGCGTCTCCACGAACAACTTCAAA	1
Alsophila spinulosa	As_target1	GCGATGGTGCCACGGTCGGCTGAAA	9
	As_target2	ACGTGCCTAGCCCGCTCGATCGAAA	11
Selaginella tamariscina	St_target1	TTTGGCCGTCCGTGGTGTTCTCTCC	23

Table S4. List of plants used to verify Genome Analysis and Genome Editing (GAGE)

Class	Family	Species	Locality	Resource type
Angiosperms	Iridaceae	*Crocus sativus*	Dingzhou, Hebei	Cultivated
Angiosperms	Compositae	*Carthamus tinctorius*	Urumqi, Xinjiang	Cultivated
Angiosperms	Nelumbonaceae	*Nelumbo nucifera*	Beijing	Cultivated
Angiosperms	Euphorbiaceae	*Ricinus communis*	Qingyuan, Guangdong	Wild
Angiosperms	Poaceae	*Setaria italica*	Suqian, Jiangsu	Cultivated
Angiosperms	Poaceae	*Zea mays*	Nanning, Guangxi	Cultivated
Gymnosperms	Ginkgoaceae	*Ginkgo biloba*	Beijing	Cultivated
Ferns	Cyatheaceae	*Alsophila spinulosa*	Meishan, Sichuan	Cultivated
Lycophytes	Selaginellaceae	*Selaginella tamariscina*	Nanning, Guangxi	Cultivated

5'-GAGAGGAGAGAGGGGTGGGTGTGAATAAGGGGAAGAGGGGGAGAGGAGGG-3'

Fig. S1. The nucleotide sequence for 50 bp ssDNA reporter[1]

Supplementary References

1. Bonini, A. et al. A label-free impedance biosensing assay based on CRISPR/Cas12a collateral activity for bacterial DNA detection. *J. Pharmaceut. Biomed.* **204**, 114268 (2021).
